# Melatonin Regulates the Neurotransmitter Secretion Disorder Induced by Caffeine Through the Microbiota-Gut-Brain Axis in Zebrafish (*Danio rerio*)

**DOI:** 10.3389/fcell.2021.678190

**Published:** 2021-05-20

**Authors:** Zeng Zhang, Qiannan Peng, Dongxue Huo, Shuaiming Jiang, Chenchen Ma, Haibo Chang, Kaining Chen, Congfa Li, Yonggui Pan, Jiachao Zhang

**Affiliations:** ^1^Key Laboratory of Food Nutrition and Functional Food of Hainan Province, College of Food Science and Engineering, Hainan University, Haikou, China; ^2^Institute of Marine Science and Technology, Shandong University, Qingdao, China; ^3^Hainan Provincial People’s Hospital, Haikou, China

**Keywords:** microbiota-gut-brain axis, intestinal microbiota, melatonin, neurotransmitter, probiotic, SCFAs

## Abstract

Melatonin has been widely used as a “probiotic agent” capable of producing strong neurotransmitter secretion regulatory effects, and the microbiota-gut-brain axis-related studies have also highlighted the role of the gut microbiota in neuromodulation. In the present study, a zebrafish neural hyperactivity model was established using caffeine induction to explore the regulatory effects of melatonin and probiotic on neurotransmitter secretion disorder in zebrafish. Disorders of brain neurotransmitter secretion (dopamine, γ-aminobutyric acid, and 5-hydroxytryptamine) caused by caffeine were improved after interference treatment with melatonin or probiotic. Shotgun metagenomic sequencing demonstrated that the melatonin-treated zebrafish gradually restored their normal intestinal microbiota and metabolic pathways. Germ-free (GF) zebrafish were used to verify the essential role of intestinal microbes in the regulation of neurotransmitter secretion. The results of the neurotransmitter and short-chain fatty acid determination revealed that the effect on the zebrafish in the GF group could not achieve that on the zebrafish in the melatonin group after adding the same dose of melatonin. The present research revealed the potential mode of action of melatonin through the microbiota-gut-brain axis to regulate the disruption of neurotransmitter secretion, supporting the future development of psychotropic drugs targeting the intestinal microbiota.

## Introduction

A good mental state, which is closely related to the level of neurotransmitter production, is indispensable for optimal human health. Caffeine is a neuroactive substance, but excessive caffeine can affect the activity of neurotransmitters (Owolabi et al., 2017). Such as down-regulating the γ-aminobutyric acid (γ-GABA) type A receptor and dopamine (DA) pathway in the hypothalamus, causing sleep disorders and increasing motor activity (Alasmari, 2020). While the cerebral neurotransmitter dysfunction can often trigger a variety of diseases, including sleep disorders, endocrine dysfunction, neurasthenia, decreased immune function and damaging health, and even causing diseases (Knutson et al., 2007; Spaeth et al., 2013). Melatonin, known as the physiological hypnagogue, is an indolyl neuroendocrine molecule secreted by the pineal gland that plays a vital role in maintaining mental health (Claustrat and Leston, 2015). Melatonin and its receptors are widely distributed in the gastrointestinal tract, with the amount of melatonin in the intestine being an estimated 400 times greater than that in the pineal gland and 10–100 times greater than that in the plasma (Bubenik, 2001; Dubocovich and Markowska, 2005). As an endogenous synchronizing factor of the circadian clock, melatonin can regulate the formation and release of neurotransmitters, such as 5-hydroxytryptamine (5-HT), γ-GABA, and DA (Acuna-Castroviejo et al., 1995; Bartell et al., 2007; Williams, 2018). Therefore, it plays a role in regulating sleep-wake biorhythm, sedation, and hypnosis via interaction on specific receptors (Keijzer et al., 2014; Gandhi et al., 2015). Present studies have indicated that melatonin is closely associated with anxiety, depression, schizophrenia, phobia, and other mental diseases (Slats et al., 2013; Bassani et al., 2014). Melatonin plays a beneficial role in intestinal dynamics and intestinal immunity, and the abundance of some butyrate-producing bacteria was positively correlated with the level of melatonin expression (Wang et al., 2021). Furthermore, melatonin can regulate the gut microbiome in mice, improve metabolic disorders (Yin et al., 2018) and reverse the effects of sleep deprivation on the intestinal microbiome in mice (Gao et al., 2019). Similarly, on the one hand, gut microbiome and its metabolites (short-chain fatty acids, SCFAs) can influence the expression of central and hepatic biological clock genes and regulate the composition of the organism through circadian transcription factors in the intestinal epithelium (Matenchuk et al., 2020). Lack of sleep, on the other hand, affects the gut microbiome by affecting food intake and reducing physical activity. The gut microbiome affects sleep by modulating serotonin (melatonin precursor) or immune pathways (O’Mahony et al., 2015; Yano et al., 2015). The interaction between the intestinal microbiota and central nervous system (CNS) at the level of specific nerves, hormones, and immunity is referred to as the microbiota-gut-brain axis (Cryan and O’Mahony, 2011). Clinical studies have indicated that there may be multiple means of communication between the brain and gut through the immune system, vagus nerves, or microbial regulation of neuroactive compounds (Strandwitz, 2018). Regarding the latter, intestinal bacteria produce SCFAs and synthesize many hormones and brain neurotransmitters, thereby impacting brain function and host behavior (Strandwitz, 2018). Investigations using probiotics provide further evidence of microbial interactions with the microbiota-gut-brain axis. Studies have shown that probiotics alter neurotransmitter secretion in the brain through the intestinal microbiota, thereby affecting the host’s mental state. For example, certain *Lactobacillus* species play an active role in modulating depression and stress-related behaviors. Research has indicated that probiotic *Bifidobacterium* may regulate the secretion of sex hormones in polycystic ovary syndrome patients through manipulating the intestinal microbiome (Bravo et al., 2011; Zhang et al., 2019). The beneficial effects of probiotics in relieving neurological diseases also suggest a role for the microbiota in these diseases.

Zebrafish (*Danio rerio*) is a highly regarded vertebrate model system with multiple unique advantages. It has a conserved nervous system structure and the vibrant behavior pattern of vertebrates, which have been studied as a model for several neurological diseases, including Alzheimer’s disease, Parkinson’s disease, and other neurological diseases (Kalueff et al., 2014). The microbiota structure of its gut is relatively simple, which is easier than in mammals to clearly explain the relevant mechanism in the analysis of the microbiota-gut-brain axis (de Abreu et al., 2019).

**FIGURE 1:**
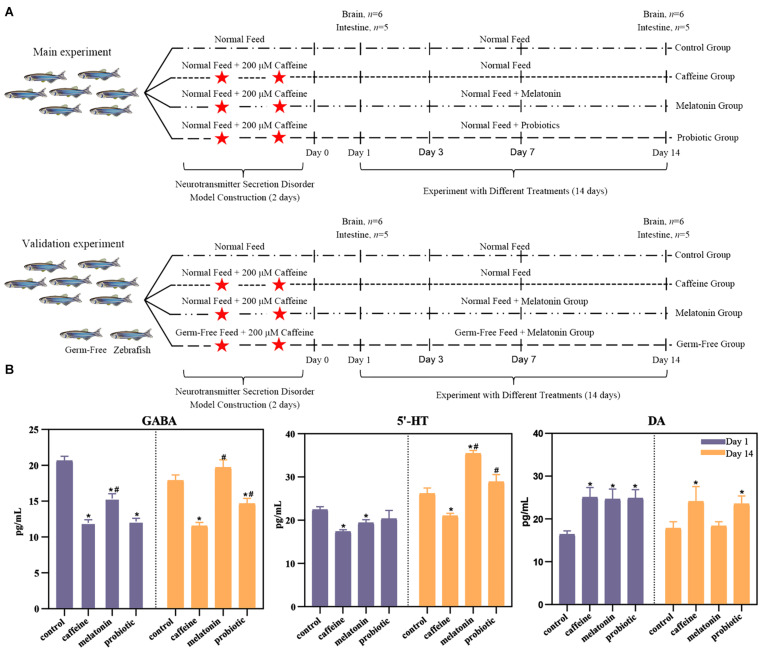
Experimental design and the effects of neural hyperactivity on the levels of neurotransmitters (DA, γ-GABA, and 5-HT) in the zebrafish brain. **(A)** Experimental design. In the main experiment, the fish were divided into four groups. The control group was treated with normal feed and regular filtered water throughout the experiment. The caffeine, melatonin, and probiotic groups were subjected to two consecutive days of caffeine exposure and then maintained under the corresponding conditions (caffeine group: normal feed + regular filtered water, melatonin group: normal feed + filtered water with 1 μM melatonin, probiotic group: feed containing 8.0 log10 *Lactobacillus plantarum* HNU082/g of feed + regular filtered water). On days 1 and 14 after caffeine induction, the brain and intestine of each zebrafish were collected for neurotransmitter level determination and high-throughput sequencing, respectively. For the verification experiment, the study added the GF zebrafish groups, and the experimental design and feed conditions were the same as the main experiment. **(B)** Effects of neural hyperactivity on the levels of neurotransmitters (DA, γ-GABA, 5-HT) in the zebrafish brain; the variation in the secretion levels of neurotransmitters in response to melatonin and probiotic supplementation were determined by ELISA. Mann-Whitney test was used to assess the differences in neurotransmitter levels in the zebrafish brain on days 1 and 14. *p < 0.05 compared with the control group. ^#^*p* < 0.05 compared with the caffeine group.

Since melatonin mitigates the effects on multiple neurological diseases, microbiota-gut-brain axis-related studies have also highlighted the role of the gut microbiota in neuromodulation. We hypothesized that the intestinal microbiota plays an important role in the improvement of neurotransmitter secretion disorders via melatonin. Additionally, we tested whether probiotics, which regulated the structure and metabolism of the intestinal microbiota, had the same effect as melatonin. To address these questions, we constructed a model of neural hyperactivity in zebrafish and modified this disorder by exogenous melatonin and probiotic administration. We utilized metagenomic approach to assess how exogenous melatonin and probiotics improve the neurotransmitter secretion disorder of zebrafish by adjusting the intestinal microbiota, which enabled us to better understand their roles in regulating neurotransmitter secretion disorders (Figure 1A). Moreover, germ-free (GF) zebrafish were also used to verify the important role of the gut microbiota in the regulation of neurotransmitter secretion disorders by melatonin. In the present research, we identified the potential mode of action through the microbiota-gut-brain axis to regulate the disruption of neurotransmitter secretion, laying a foundation for exploring the prevention and treatment of some neuropsychiatric disorders by improving the intestinal microbiota.

## Materials and Methods

### Experimental Design

In experiment 1, fish were equally distributed into 24 glass tanks (15 fish/tank) after 14 days of acclimation, each containing 5 L of water. Then, tanks were randomly assigned to four groups to experiment (Figure 1A): (1) control group: normal feed, regular filtered water; (2) caffeine group: 200 μM caffeine was added to the water for two consecutive days to alter zebrafish activity. Thereafter the zebrafish were maintained conventionally with standard feed and regular filter water; (3) melatonin group: 200 μM caffeine was used for two consecutive days to change zebrafish activity, and then the fish were fed regularly, but the water was replaced with filtered water containing 1 μM melatonin (Shanghai Macklin Biochemical Co., Ltd., Shanghai, China), melatonin powder was dissolved in anhydrous ethanol to 100 mM and eventually diluted to a final concentration of 1 μM); and (4) probiotic group: zebrafish were treated with 200 μM caffeine for two consecutive days to change the activity of fish. Then they were maintained in regular filtered water *Lactobacillus plantarum* HNU082 cells were pelleted by centrifugation and resuspended in 50 μL of saline, which was mixed with feed at 8.0 log10 CFU/g diet (Ma et al., 2020). Unconsumed food, feces, and dead fish were removed on time, and the corresponding fresh filtered water was replaced in each tank every day; the water for the melatonin group was replaced with filtered water containing 1 μM melatonin to maintain the constant melatonin concentration, while the water for the other three groups was replaced with regular filtered water.

To verify the critical role of the intestinal microbiota in the secretion of related neurotransmitters, GF zebrafish were also studied (Figure 1A). The zebrafish were purchased from the China Zebrafish Resource Center (CZRC), and the GF zebrafish model was established as previously reported (Ding et al., 2018). Considering that the effects of neurotransmitter regulation were best in the melatonin-treated fish, we used only four groups, the control, caffeine, GF, and melatonin groups. The rearing and treatment conditions of the control, caffeine, and melatonin groups were the same as those in the experiment above. Two-day caffeine induction was conducted, and melatonin supplementation was performed for 14 days, as in the other treatment groups. Water and feed were sterilized by irradiation with ultraviolet radiation in advance. On days 1 and 14 after 2-day caffeine induction in experiment 2, the mean static time of the fish school in 5 min under night-time dark conditions in the control, caffeine, melatonin, and GF groups were observed and recorded same as the first experiment.

### Zebrafish Maintenance and Feeding

Adult zebrafish (including male and female adults, 3 months old, average body length 3.2 ± 0.1 cm) obtained from the CZRC were maintained in the aquatic experimental animal facility of the College of Food Science and Engineering, Hainan University. The experimental and animal care procedures were conducted following the recommendations of the Ethics Committee of Hainan University. All fish were housed for 14 days in 50 L tanks supplied with aerated and dechlorinated tap water to acclimate to the laboratory conditions before experiments. The acclimation period and experiments were performed at a temperature of 28 ± 0.5°C and with a 14 h light/10 h dark cycle according to the standards of zebrafish care. The water was changed and the fish tank was cleaned every morning. Commercial fodder (Sanyou Chuangmei, China) was provided once daily at a fixed time, which was administered by 3% of the total weight of the fish in each tank. Dead zebrafish were removed promptly, and the cumulative mortality did not exceed 2% throughout the acclimation period. For the GF group, whole tanks were kept in the clean bench ventilated with filtered sterile air to maintain germ-free conditions until the end of the experiment. Sterile food was performed through ultraviolet sterilization. Water was treated by the filter-sterilized system and irradiated overnight with ultraviolet in the clean bench to keep sterilize and ensured that the temperature was maintained at room temperature during morning transfer.

### Sample Collection for 16S rRNA and Shotgun Metagenomic Sequencing, SCFAs Determination, and ELISA

Several zebrafish were randomly selected from each group on days 1, 3, 7, and day 14 following the 2-day caffeine induction. Fish were euthanized promptly by an ice water bath, and dissection was conducted vice the anus with an aseptic scalpel after the body was sterilized with 75% alcohol. The entire intestine from the esophagus to the anus was collected, and three fish were pooled as a sample, taking a total of 6 replicates in each group for DNA extraction and a total of 3 replicates in each group for short-chain fatty acid (SCFA) determination. After centrifugation at 13,198 × g for 20 min at 4°C, the head supernatants were subsequently collected and stored at −80°C for ELISA. In the verification experiment, another three were collected as a sample, with a total of three replicates in each group, to conduct gene expression analysis by real-time PCR.

### SCFAs Analysis

The fatty acid concentrations in zebrafish intestines were quantified by a gas chromatography-mass spectrometry (GC-MS) assay (Wei et al., 2015). Sample preparation were prepared according to the method of Ma et al. (2020). 2 mL of H_2_SO_4_ (0.5 mol/L) was added after the samples were weighed. Then, they were extracted by ultrasonic shaking (40°C, 35 kHz) for 30 min. After that, 1 mL of diethyl ether was added and the samples were kept at 4°C for 5 min and centrifuged (12,000 × g, 5 min). Finally, 100 μL of acetone was added for GC-MS analysis after diethyl ether was removed. An Agilent 7890B gas chromatograph equipped with a mass spectrometer (Agilent 5977A, Agilent, Santa Clara, CA, United States) was used to analyze samples, and an HP-5 MS column (30 m × 0.25 mm, 0.25 μm, Agilent, Santa Clara, CA, United States) was employed to achieve separation. The oven program was as follows: 50°C for 1 min, rise to 200°C at a rate of 10°C/min, held at 200°C for 5 min, rise to 220°C at a rate of 5°C/min, held at 220°C for 10 min, rise to 250°C at 15°C/min, and held at 250°C for 10 min. The temperature of the inlet was held at 250°C, and the mass range was scanned from *m/z* 35 to 400. The temperature of the ion source chamber and the transfer line were set at 230 and 250°C, respectively, with an electron energy of 70 eV.

### Enzyme-Linked Immunosorbent Assay (ELISA)

After the samples were thawed, they were used for assessment by ELISA kits (Shanghai Xin Yu Biotech Co., Ltd., Shanghai, China) according to the manufacturer’s instructions. The concentrations of DA, γ-GABA, and 5-HT in tissue were calculated based on the optical absorbance value at 450 nm (OD450) determined by a microplate reader (SpectraMax M2, MD, Shanghai, China). All samples were tested three times and the average was taken as the result.

### Real-Time PCR for Gene Expression Analysis

The total RNA was isolated according to the manufacturer’s instructions. A High-Capacity cDNA Reverse Transcription Kit (Applied Biosystems, CA, United States) was used to conduct cDNA synthesis with the entire isolated RNA following the instructions. Real-time PCR was performed to determine the mRNA levels of the genes. The PCR mixtures (20 μl) contained 10 μl of SYBR green mix (TAKARA, Japan), 0.2 μM each forward and reverse primer and, 1 μl of cDNA. The primers used refer to the preparation of previous studies (Borrelli et al., 2016; Ding et al., 2018; Teng et al., 2018; Moriya et al., 2019). A StepOne Plus System (Applied Biosystems) was used to conduct the real-time PCR under the condition of 10 min at 95°C, followed by 40 cycles at 95°C for 15 s and 1 min at 60°C, and ended by a dissociation step. StepOne Plus software was used to analyze of the results. The relative mRNA levels of genes were quantified according to the 2^–Δ^ ^Δ^ ^*Ct*^ method (Livak and Schmittgen, 2001). Details of the primers were given in Supplementary Table 1.

### DNA Extraction From Zebrafish Intestine Tissues and 16S Ribosomal RNA Gene Sequencing

Genomic DNA of zebrafish intestine was extracted using a QIAamp^®^ DNA Mini Kit (Qiagen, Hilden, Germany) following the manufacturer’s instructions. The quality of all isolated DNA was assessed by A260/A280 using a NanoDrop instrument. Qualified DNA was preserved at −20°C for subsequent analysis. The PCR amplification procedure was conducted as previously described (Dethlefsen and Relman, 2011). A set of 6-nucleotide barcodes was added to the specific primers 338F and 806R to amplify the 16S rRNA V3-V4 region of the genomic DNA (Ma et al., 2020). Details of the primers were given in Supplementary Table 1. An Agilent DNA 1000 kit combined with an Agilent 2100 Biologic Analyzer (Agilent Technologies, United States) was used to quantify the PCR products. Sequencing was performed using the Illumina MiSeq high-throughput sequencing platform by Shanghai Personal-Bio Corporation.

### Shotgun Metagenomic Sequencing and Quality Control

The zebrafish samples on day 14 (*n* = 20, 5 samples each group) were subjected to shotgun metagenomic sequencing by using an Illumina HiSeq 2500 instrument. Libraries were prepared with a fragment length of approximately 300 bp. Paired-end reads were generated using 100 bp in the forward and reverse directions. The reads were trimmed using Sickle and subsequently aligned to the zebrafish genome to remove the host DNA fragments (Zhang et al., 2020).

### Functional Annotation and Metabolic Pathway Analysis

The annotated amino acid sequences were aligned against the Kyoto Encyclopedia of Genes and Genomes (KEGG) databases using BLASTp (*e* ≤ 1e-5 with a bit-score higher than 60). The annotated sequences were assigned to the KEGG ortholog group (KO) according to the highest score. Reporter Z-scores were calculated to reveal the differences in enriched metabolic pathways between the control and PCOS groups, as previously described. Accordingly, a reporter score of > 2.3 (90% confidence according to the normal distribution) was used as a detection threshold to significantly differentiate between pathways (Qin et al., 2014).

### Bioinformatic Analyses and Statistical Analyses for Figure Construction

A quality-control procedure was performed for the raw pair-end reads; bioinformatics analysis of high-quality sequence data was conducted after removal of primer and barcode sequences by using the QIIME platform (Caporaso et al., 2010b). PyNAST (Caporaso et al., 2010a) was used to align the trimmed sequences, and those under 100% sequence identity were clustered using UCLUST (Edgar, 2010) to obtain a unique V3-V4 sequence set. On this basis, operational taxonomic units (OTUs) were clustered at a threshold level of 97% sequence identity, and the sequences with the highest frequency were selected as the representative sequences of OTUs. After ChimeraSlayer (Haas et al., 2011) was employed to remove the potentially chimeric sequences in the representative set of OTUs, a representative sequence of each OTU was assigned to a taxonomic level by Ribosomal Database Project (RDP) (Cole et al., 2007). A phylogenetic tree of OTUs was constructed by using Fast Tree (Price et al., 2009), and the alpha and beta diversity of bacterial communities were analyzed on this basis. The sequence data reported in this paper have been deposited in the NCBI database (metagenomic data: PRJNA543612).

Relatedness analysis was conducted with R Software. Boxplot based on weighted and unweighted UniFrac distance was established using the ggplot2 package. Principal component analysis (PCA) was carried out by the ggord package, and the PHEATMAP package was used for cluster analysis and heatmap construction. Correlation analysis was calculated by using Spearman’s rank correlation coefficient and visualized as a network by Cytoscape. Statistical significance was analyzed with Mann-Whitney test or *T*-test. Data were presented as the means ± SEMs; a two-sided test was conducted, and *p* < 0.05 was considered statistically significant. All statistical analyses were performed by GraphPad Prism (version 8) and R (version 3.6.1).

## Results

### Alteration of Neurotransmitters in the Zebrafish Brain

To investigate the effects of different treatments on neurotransmitter secretion, the levels of neural hyperactivity-related neurotransmitters in each group were determined (Figure 1B). On day 1, the concentration of DA increased significantly in the caffeine, melatonin, and probiotic groups, and γ-GABA concentrations decreased significantly. The concentration of 5-HT was significantly decreased in the caffeine and melatonin groups. There was also a decrease in the probiotic group although there was no significant difference. The imbalance indicated that caffeine had successfully induced neurotransmitter secretion disorders in the zebrafish brain. After 14 days of treatment in different experimental groups, the level of the neurotransmitters in each group changed. In the caffeine group, the concentrations of the three neurotransmitters were not well recovered and remained significantly different from the control group. On the contrary, the concentrations of γ-GABA and 5-HT were increased, and DA was decreased in the melatonin group. The concentrations of all three neurotransmitters were restored to the levels of the control group. Interestingly, the concentrations of γ-GABA and 5-HT were even significantly higher than those in the caffeine group. In the probiotic group, the concentration of γ-GABA was significantly higher than in the caffeine group, although still lower than in the control group. The concentration of 5-HT was elevated and significantly higher than in the caffeine group. While the concentration of DA was somewhat reduced, although there was still a significant difference compared to the control group.

### Effects of Different Treatments on Altered Zebrafish Intestinal Microbiota

**FIGURE 2:**
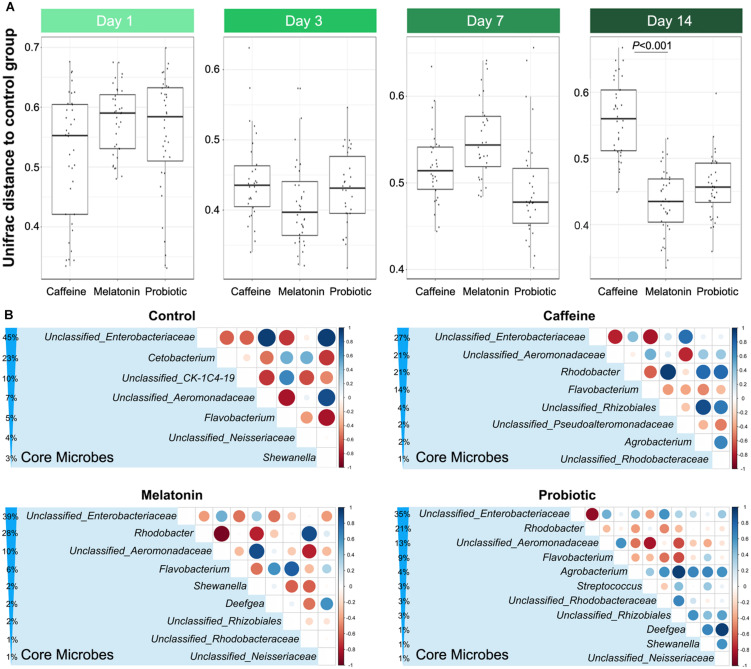
Comparison of similarities and core genera between groups. **(A)** The distance based on the unweighted UniFrac between the control and three different treatment groups displays the difference of the gut microbiota among different groups on days 1, 3, 7, and 14, which include control vs. caffeine, control vs. melatonin, and control vs. probiotic. **(B)** Comparative analysis of core genera accounting for more than 1% in the control, caffeine, melatonin, and probiotic groups after 14 days of corresponding treatment. The percentage indicates the relative abundance of the corresponding genus. The size and color of the circle are proportional to the correlation intensity. The redder the color, the stronger the negative correlation, and the bluer the color, the stronger the positive correlation.

The distance based on unweighted UniFrac in Figure 2A showed that the distances between the three treatment groups and the control group remained at similar levels after the caffeine interference. After 14 days of treatment, the similarity between the melatonin and control groups was significantly higher than that of the caffeine group, although there was no significant difference in the weighted UniFrac distance (Supplementary Figure 1). PCA presented in Supplementary Figure 2 also demonstrated that the points representing caffeine, melatonin, and probiotic groups were highly disordered but different from the control group on day 1, which indicated that the intestinal microbiota of zebrafish was disturbed by caffeine. After 14 days of corresponding treatment, the microbial community structure in each treatment group was like that in the control group after 14 days of treatment, the melatonin group had the highest similarity with the control group, followed by the probiotic and caffeine groups. Taken together, these data suggested that caffeine induction results in some destabilization in the zebrafish microbial community, while melatonin and probiotic supplementation were able to draw closer the similarity between the treatment and control groups.

Based on the high-throughput 16S rRNA sequencing data, the effects of different treatments on the gut microbiota of zebrafish were evaluated, and the alpha diversity of the zebrafish intestinal microbiota in different groups were compared on days 1 and 14 (Supplementary Tables 2, 3). On the first day, there was no significant difference among the groups. After 14 days of equal treatment, the Chao1, ACE, Shannon, and Simpson indices in the caffeine group were significantly higher than those in the control group. Moreover, supplementation with melatonin slightly decreased intestinal microbial diversity compared with that in the caffeine group, while that in the probiotic group remained at a high level. Since different treatments had specific effects on the intestinal microbiota of zebrafish, we next selected the core genera accounting for more than 1% in each group after 14 days of treatment for comparative analysis (Figure 2B). Of the seven core microbes in the control group, five genera were identical to the melatonin and probiotic groups, while only three genera were identical to the caffeine group in the control group. This result implied that melatonin and probiotic can regulate the core microbiota of the zebrafish gut to some extent and toward the control group.

**FIGURE 3:**
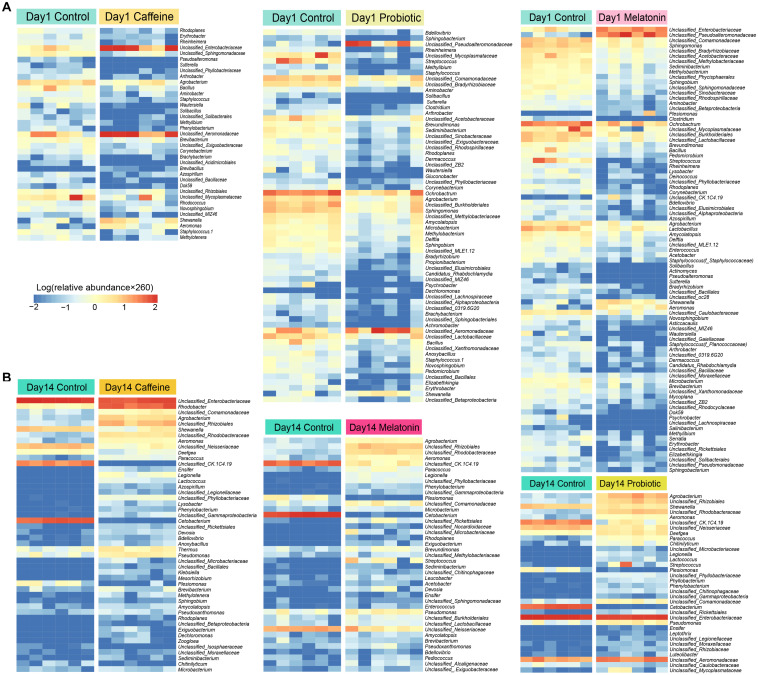
Screening of differential genera in the caffeine group, melatonin group, and probiotic group compared with the control group on days 1 and 14. **(A)** The significant difference genera were screened in the caffeine group, melatonin group, and probiotic group compared with the control group on day 1. **(B)** The significant difference genera were screened in the caffeine group, melatonin group, and probiotic group compared with the control group on day 14. The depth degree of color represents the relative abundance of the genus or species (The blue indicates a small number and the red indicates a large number). Mann-Whitney test was used on days 1 and 14.

Accordingly, we identified the differential genera among the three treatment groups (the caffeine group, the probiotic group, and the melatonin group) compared with the control group on day 1 (Figure 3A) and day 14 (Figure 3B). On day 1, there were a certain number of different genera in three treatment groups compared with the control group. The abundance of genera such as *Mycoplasmatacea*, *Bacillus*, and *Arthrobacter* decreased significantly, while *Aeromonadaceae* and *Shewanella* increased significantly. After 14 days of different treatments, the relative abundance of *Shewanella* decreased significantly in all three groups. The difference of *Aeromonadaceae* between the melatonin group and the control group disappeared. However, its relative abundance in the caffeine group and the probiotic group was still significantly higher than that in the control group. More importantly, the number of differential genera was reduced in the melatonin group and the probiotic group, compared with the control group. These results suggested that melatonin supplementation gradually modified the difference of intestinal genera in zebrafish induced by caffeine. Collectively, caffeine induction results in some destabilization in the zebrafish microbial community, while melatonin and probiotic supplementation regulated this effect.

### Functional Features of the Intestinal Microbiota in Different Treatments

**FIGURE 4:**
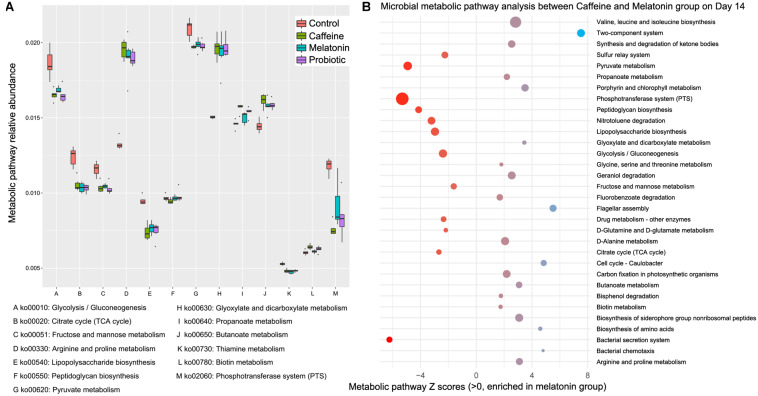
Differential functional features of the zebrafish intestinal microbiota in the caffeine, melatonin, probiotic, and control groups after 14 days of corresponding treatment. Metabolic pathway analysis was performed based on the shotgun metagenomic sequencing data, which were compared with the KEGG database to further explain the microbial metabolic pathways. **(A)** Metabolic pathway relative abundance. **(B)** Microbial metabolic pathway analysis between Caffeine and Melatonin group on day 14, Z scores >1.6 were selected as significant differences.

To investigate how caffeine interference and the supplementation of melatonin and probiotic effect metabolic pathways by regulating intestinal microbiota, we performed shotgun metagenomic sequencing for samples collected on day 14 (*n* = 20). We annotated the assembled genes and compared with KEGG database to further annotate the microbial metabolic pathways. The Z score >1.6 represented the significant differences. The results showed that different treatments significantly altered the metabolism of zebrafish intestinal microbiota (Figure 4 and Supplementary Figure 3). Compared with normal zebrafish, arginine and proline metabolism (ko00330), glyoxylate and dicarboxylate metabolism (ko00630), propanoate metabolism (ko00640), butanoate metabolism (ko00650), and biotin metabolism (ko00780) were enriched in the caffeine group. Glycolysis/gluconeogenesis (ko00010), citrate cycle (ko00020), fructose and mannose metabolism (ko00051), lipopolysaccharide biosynthesis (ko00540), pyruvate metabolism (ko00620), thiamine metabolism (ko00730), and phosphotransferase system (ko02060) were enriched in the control group. Collectively, the caffeine induction, melatonin and probiotic supplementation markedly altered the metabolism of the zebrafish gut microbiota.

### Verification Experiment Through a GF Zebrafish Model

**FIGURE 5:**
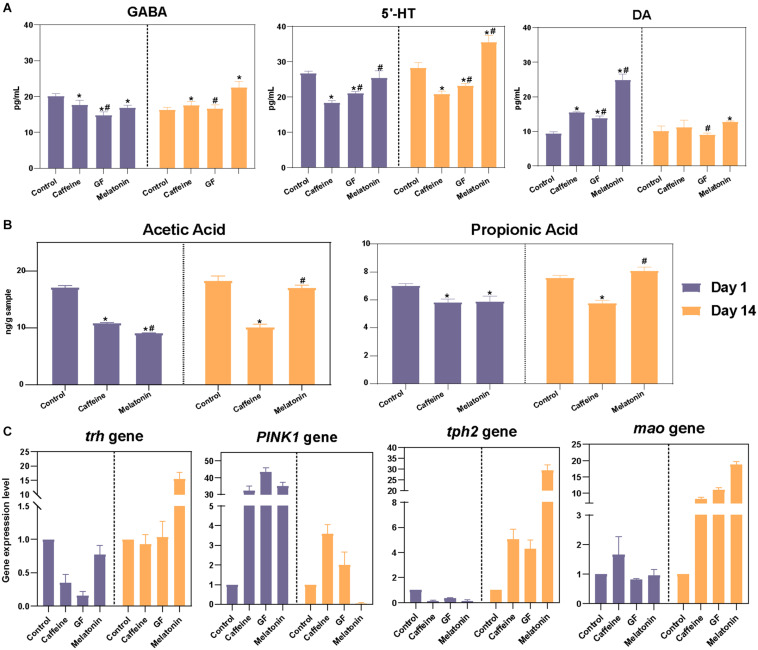
Correlation analysis of neurotransmitter levels in validation experiments. **(A)** Further verification was performed using germ-free zebrafish, and the levels of neurotransmitters in the zebrafish brain, including DA, γ-GABA, and 5-HT, were detected on days 1 and 14 among the control, caffeine, GF, and melatonin groups. **(B)** GC-MS analysis of intestinal acetic acid and propionic acid concentrations in zebrafish and contents are expressed as nanograms per gram of intestine. **(C)** The zebrafish genes associated with DA (*PINK1*), γ-GABA (*trh*), and 5-HT (*tph2* and *mao*) secretion were selected and subjected to real-time PCR analysis.

To verify that the gut microbiota played an essential role in the function of melatonin, a GF zebrafish model was used. Considering that the effects of neurotransmitter regulation were best in the melatonin group (Supplementary Figure 4B, up panel), we only retained four groups: the control, caffeine, GF, and melatonin groups. Compared with the results of the control group, the caffeine interference increased the hyperactivity and decreased the rest time of the zebrafish. Moreover, after adding the same dose of melatonin, the rest time which could reflect brain activity was significantly different between the GF and melatonin groups (Supplementary Figure 4B, bottom panel), a better effect was observed in the melatonin group. However, we did not observe any significant difference in the phenotype including body weight and body length among different groups (Supplementary Figures 4A,C). Correspondingly, the neurotransmitter secretions γ-GABA, 5-HT, DA, and their related synthetic genes were also found to be different (Figures 5A–C). This confirmed the primary mediate role of the intestinal microbiota in the efficacy of melatonin. Notably, from both two experiments, we observed the disorders of brain neurotransmitter secretion caused by caffeine, including that of DA, γ-GABA, and 5-HT, were improved after interference treatment with melatonin. However, some discrepancies in the specific value of the same group were observed between the 2 experiments because of the zebrafish individual heterogeneity. Furthermore, 4 zebrafish genes related to the secretion of γ-GABA, 5-HT, and DA were selected for gene expression analysis (*PINK1*, corresponding to DA; *trh*, corresponding to γ-GABA; and *tph2* and *mao*, corresponding 5-HT) showing high consistency with the results of zebrafish rest time, as shown in Figure 5C.

To better explain the role by which the intestine microbiota function in the process of melatonin regulation of neurotransmitter secretion disorders, we also determined the contents of metabolites in the gut of zebrafish. Since GF zebrafish had almost no intestinal microbiota, while the SCFAs were mainly produced by colonic anaerobic fermentation of undigested carbohydrates, SCFAs were not detected in the GF group here. Results showed that the contents of acetic acid and propionic acid in the intestine of zebrafish in the caffeine and melatonin groups decreased significantly after the treatment with caffeine, while the contents increased remarkably after melatonin treatment, resulting in levels that were obviously higher than those in the caffeine group (Figure 5B).

### Melatonin Regulated Neurotransmitter Secretion Disorders Through the Microbiota-Gut-Brain Axis

**FIGURE 6:**
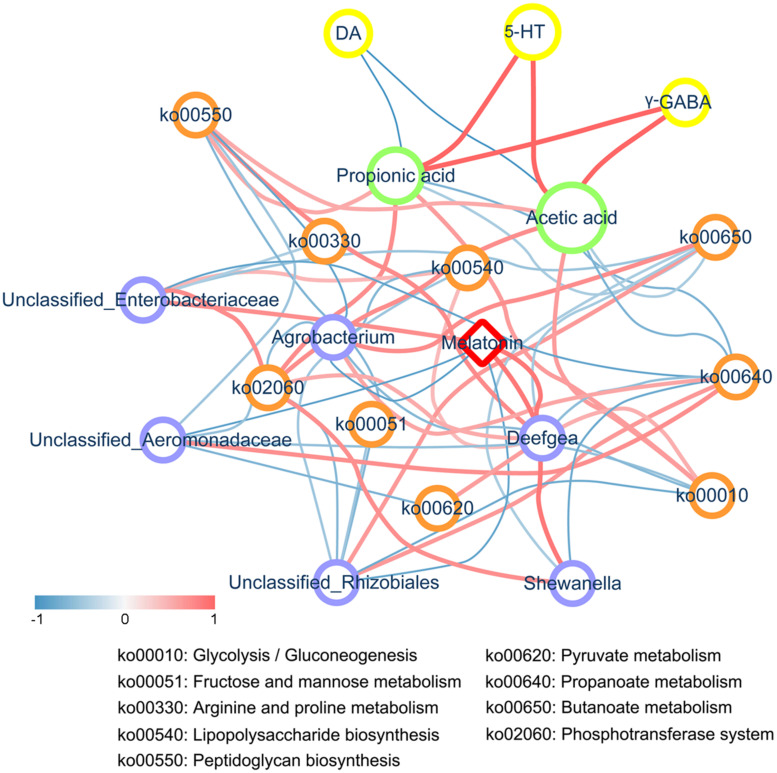
Correlation network analysis. The correlation network constructed among melatonin, differentially abundant genera, metabolic pathways, SCFAs, and neurotransmitters (including DA, γ-GABA, and 5-HT) based on Spearman’s rank correlation coefficient. An *R*< -0.4 or > 0.4 was selected. The width and color (the red indicates a positive correlation, while the blue indicates a negative correlation) of the edge are proportional to the correlation intensity. The red node represents melatonin, blue nodes represent genera, orange nodes represent metabolic pathways, green nodes represent SCFAs, and yellow nodes represent neurotransmitters. The node size is proportional to the mean abundance in the respective population.

Through the verification experiment, we highlighted the importance of intestinal microbiota in the melatonin-induced neurotransmitter regulation effects. Based on the Spearman’s rank correlation coefficient, we constructed a network of the correlations among melatonin, genera, metabolic pathways, SCFAs, and neurotransmitters. As shown in Figure 6, supplementation with melatonin inhibited the growth of *Shewanella*, *Deefgea*, and *Enterobacteriaceae* in the zebrafish gut, which were negatively correlated with propanoate metabolism, butanoate metabolism, and phosphotransferase system, whereas a negative correlation was observed between *Aeromonadaceae* and *Rhizobiales* and melatonin. Additionally, it was found that melatonin supplementation elevated the levels of acetic acid and propionic acid, representing SCFAs, in the intestine, and the generated SCFAs further stimulated the secretion of neurotransmitters in the brain of zebrafish, accompanied by decreased levels of DA and elevated levels of γ-GABA and 5-HT, which indicated that melatonin could regulate the disorder of neurotransmitter secretion through the microbial-gut-brain axis.

## Discussion

In addition to the variation in the levels of neurotransmitters, the structure of the zebrafish gut microbiota of zebrafish was disordered after caffeine induction. After 14 days of treatment, the intestinal microbiota in the various groups gradually recovered, among which the similarity of the melatonin group and control group microbiota was the highest, while the caffeine group microbiota still had a large difference. Interestingly, *Aeromonas* has been shown to be associated with bacterial septicemia and other bacterial fish diseases (Qin et al., 2017; Wu et al., 2018). The abundance of *Aeromonadaceae* in the melatonin group decreased significantly after 14 days of treatment and the difference between it and the control group disappeared. However, its relative abundance in the caffeine and probiotic groups remained significantly higher than in the control group. Overall, melatonin supplementation may restore the original intestinal microbiota structure to a great extent. We also observed some genus, amino acid metabolism, carbohydrate metabolism, cofactor and vitamin metabolism, and membrane transport changed significantly in different groups. Current research has indicated that SCFAs are implicated in a range of neuropsychiatric disorders, with the ability to cross the blood-brain barrier and are critical substances that connect the intestine to the central nervous system (Dalile et al., 2019). The metabolites of SCFAs, such as propanoate and butanoate, were found to be stimulated during self-healing to restore the neurotransmitter secretion levels in the caffeine group. Previous studies have shown that SCFAs can promote the production of colonic 5-HT through their effects on enterochromaffin cells (Reigstad et al., 2015), especially propanoate, which has been shown to alter the DA, 5-HT, and γ-GABA systems (El-Ansary et al., 2012). Furthermore, 5-HT activates the vagus nerve. Additionally, 5-HT is the precursor of melatonin, which may be a means to connect the brain to the intestinal microbes. Nevertheless, the SCFA metabolism in the melatonin and probiotic groups was significantly lower than that in the caffeine group. We hypothesized that supplementation might promote the production of SCFAs during neural hyperactivity regulation, which accelerates the recovery of neurotransmitter secretion in the brain and gradually regulates the hyperactive state. Therefore, the metabolism of SCFAs gradually tended toward normal in the melatonin and probiotic groups.

GF animals were used to verify whether gut microbes actually play a real role in the recovery of brain neurotransmitter secretion. This is a valid and frequently used *in vivo* experimental model for studies on the host gut microbiota in health and various diseases, such as cancer (Pollard and Sharon, 1974), cardiovascular (Wang et al., 2011) and metabolic diseases (Nicholson et al., 2012). As the importance of the microbiota-gut-brain axis in brain development has gradually been confirmed, GF animals have gradually become an accepted model for microbiota-gut-brain axis studies. In our validation experiment, the recovery of neurotransmitters in the GF group did not reach the level as in the melatonin group, indicating the key role of the intestinal microbiota in the regulation process of neurotransmitter secretion by melatonin. This conclusion was also reconfirmed in the subsequent real-time PCR. In order to better explain the role of intestinal microbiome in the regulation of neurotransmitter secretion, the content of SCFAs in the gut of zebrafish was determined. SCFAs have been shown to interact with G protein-coupled receptors or histone deacetylases to affect mental function (Correa et al., 2016). They can act directly or indirectly on the brain via humoral, hormonal, immune, and neural pathways (Dalile et al., 2019). The results showed that the contents of acetic acid and propionic acid decreased in the intestine of tested fish after caffeine induction, while the content increased after melatonin treatment and was higher than that in the fish school without melatonin treatment, corresponding to our previous speculation that melatonin may promote the secretion of neurotransmitters in the brain by affecting intestinal microbes and producing SCFAs.

## Conclusion

Collectively, melatonin supplementation recovered normal homeostasis and metabolism of the gut microbiome, which promoted the production of SCFAs and ultimately accelerates the restoration of neurotransmitter secretion levels through the microbial-gut-brain axis. This study revealed the potential mode of action of melatonin through the microbiota-gut-brain axis to regulate the disruption of neurotransmitter secretion, which supported the future development of psychotropic drugs targeting the intestinal microbiota.

## Data Availability Statement

The authors declare that the data supporting the findings of this study are available within the paper and its additional files. The sequence data reported in this manuscript have been deposited in the NCBI database (metagenomic data: PRJNA543612).

## Ethics Statement

The animal study was reviewed and approved by the Ethics Committee of Hainan University.

## Author Contributions

YP and JZ conceived and designed the experiments. JZ, QP, CL, ZZ, DH, CM, SJ, and HC performed the experiments and analyzed the data. KC, QP, JZ, and YP wrote and revised the manuscript. All authors read and approved the final manuscript.

## Conflict of Interest

The authors declare that the research was conducted in the absence of any commercial or financial relationships that could be construed as a potential conflict of interest.
