# Supplementary material for: Melatonin Regulates the Neurotransmitter Secretion Disorder Induced by Caffeine Through the Microbiota-Gut-Brain Axis in Zebrafish (Danio rerio)
Source: Front Cell Dev Biol. 2021 May 20;9:678190. doi: 10.3389/fcell.2021.678190 (PMC8172981; doi:10.3389/fcell.2021.678190)
Supplement: Supplementary file 1 [file Data_Sheet_1.PDF]

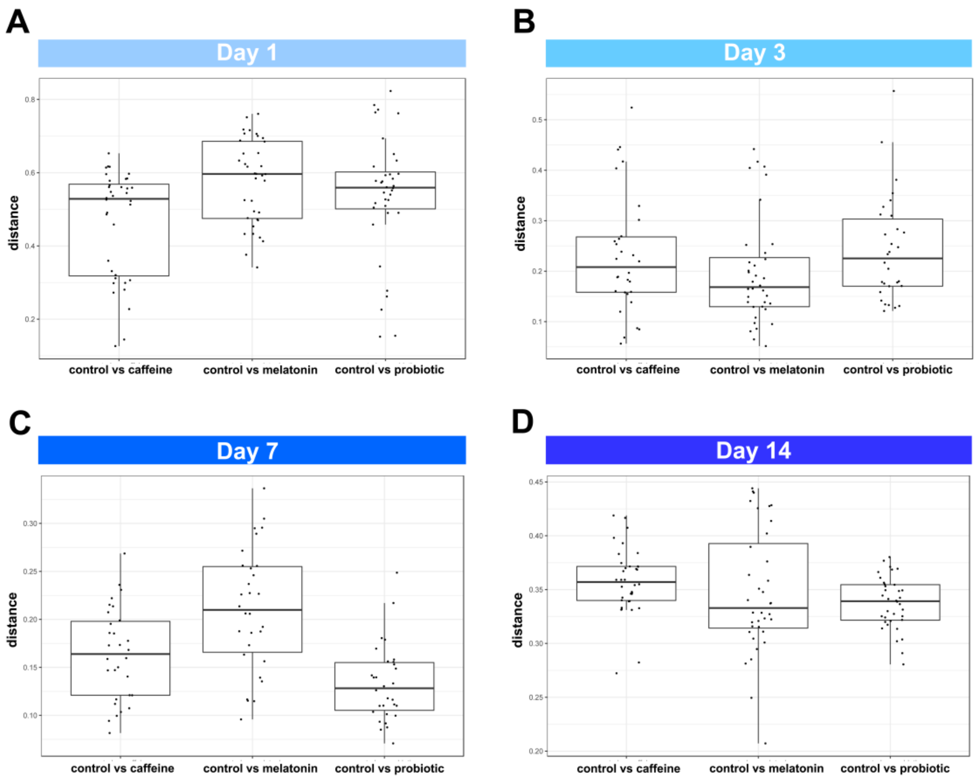

Figure S1. The distance based on the weighted unifrac between the control and three different treatment groups display the difference of the intestinal microbiota among different groups on day 1 (A), day 3 (B), day 7 (C) and day 14(D), which include control vs caffeine, control vs melatonin, and control vs probiotic.
