# Supplementary material for: Melatonin Regulates the Neurotransmitter Secretion Disorder Induced by Caffeine Through the Microbiota-Gut-Brain Axis in Zebrafish (Danio rerio)
Source: Front Cell Dev Biol. 2021 May 20;9:678190. doi: 10.3389/fcell.2021.678190 (PMC8172981; doi:10.3389/fcell.2021.678190)
Supplement: Supplementary file 2 [file Data_Sheet_2.PDF]

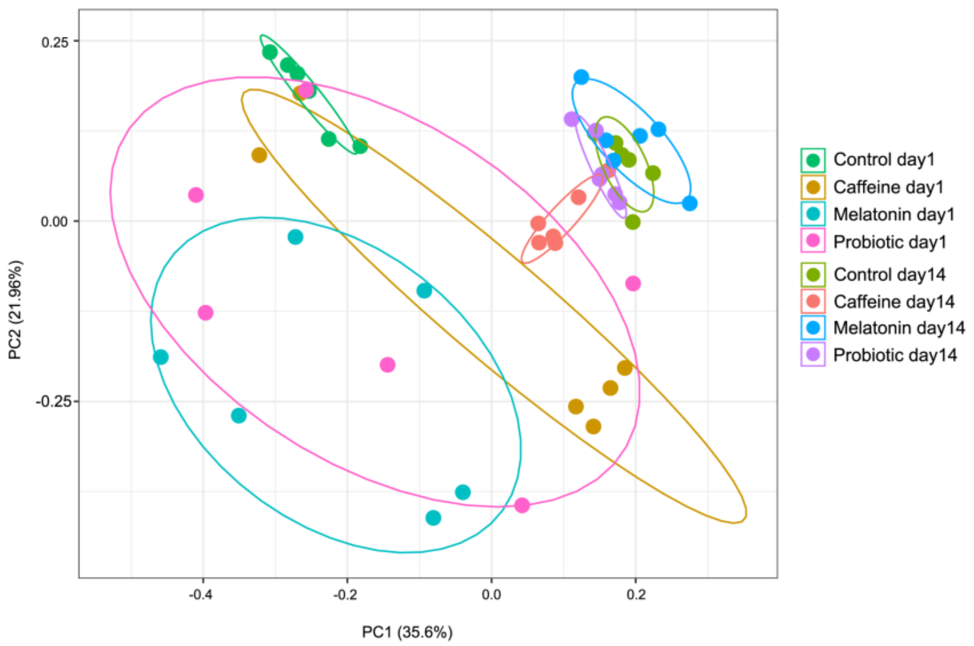

Figure S2. PCA based on the OTU in the gut. Each dot represents the composition of the gut microbiota of a sample, and different color represent zebrafish gut samples at different time points and groups.
