# Supplementary figures and images for: Melatonin Regulates the Neurotransmitter Secretion Disorder Induced by Caffeine Through the Microbiota-Gut-Brain Axis in Zebrafish (Danio rerio)
Source: Front Cell Dev Biol. 2021 May 20;9:678190. doi: 10.3389/fcell.2021.678190 (PMC8172981; doi:10.3389/fcell.2021.678190)

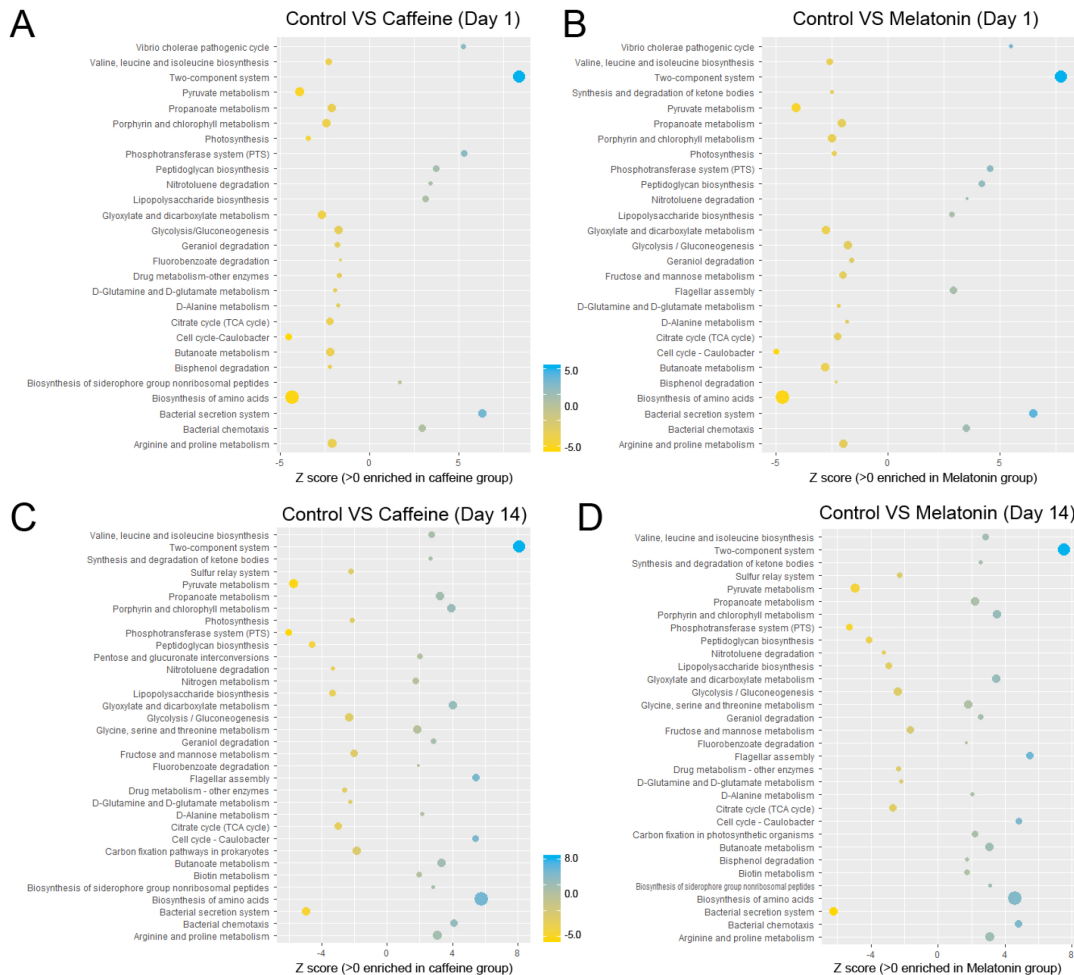

**Figure S3.** Functional features of the intestinal microbiota in different treatments.

Supplement: Supplementary file 3 [file Data_Sheet_3.pdf]
