# Supplementary material for: Melatonin Regulates the Neurotransmitter Secretion Disorder Induced by Caffeine Through the Microbiota-Gut-Brain Axis in Zebrafish (Danio rerio)
Source: Front Cell Dev Biol. 2021 May 20;9:678190. doi: 10.3389/fcell.2021.678190 (PMC8172981; doi:10.3389/fcell.2021.678190)
Supplement: Supplementary file 4 [file Data_Sheet_4.pdf]

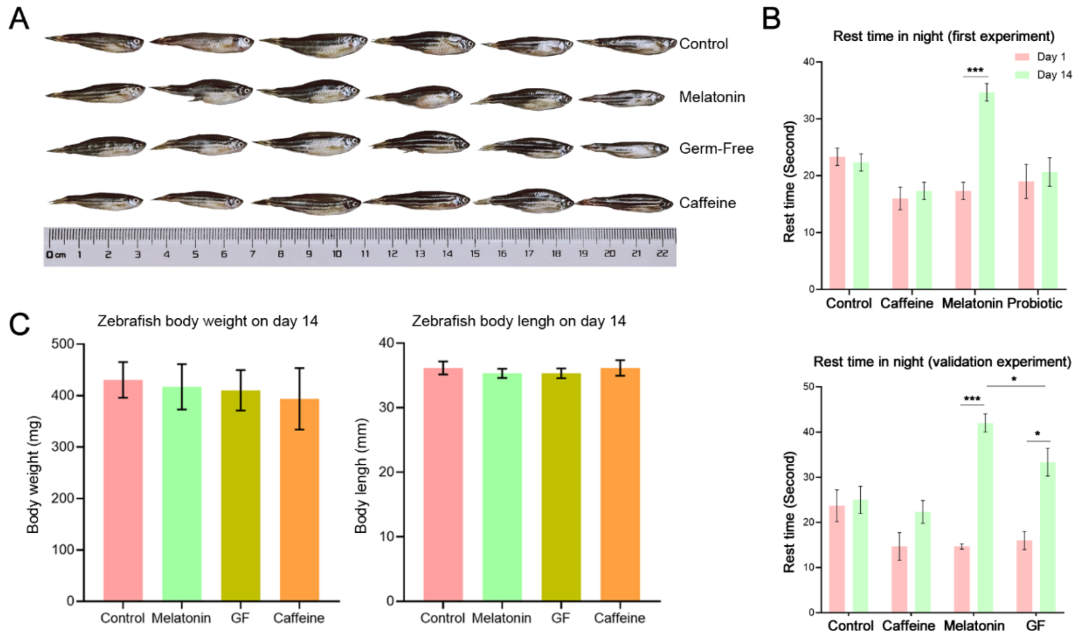

Figure S4. The performance in phenotype including (A, C) fish body weight, fish body length and (B) the rest of time in the night per 5 minutes among the groups in the first (up panel) and the verification experiment (bottom panel) on day 14. *, $p<0.05$; ***, $p<0.001$.
