# Supplementary material for: Melatonin Regulates the Neurotransmitter Secretion Disorder Induced by Caffeine Through the Microbiota-Gut-Brain Axis in Zebrafish (Danio rerio)
Source: Front Cell Dev Biol. 2021 May 20;9:678190. doi: 10.3389/fcell.2021.678190 (PMC8172981; doi:10.3389/fcell.2021.678190)
Supplement: Supplementary file 5 [file Table_1.DOCX]

**Table S1.** Primers used for 16s rRNA sequencing and real-time PCR

| Gene | Primer (5’-3’) |
| --- | --- |
| *16s rRNA (V3-V4 region)* | ACTCCTACGGGAGGCAGCA  GGACTACHVGGGTWTCTAAT |
| *tph2* | CAAGAGACAACAGCAACTATG  AAGCCCAACAGGTGATTTAG |
| *mao* | GCAGTCAGAGCCCGAATC  CACACCCATAAACTTGAGGAATC |
| *trh* | CACACAGATGGAGGAGCAGA  AGCAGCATCAGGTAGCGTTT |
| *actb1(Housekeeping gene)* | AGAGCTATGAGCTGCCTGACG  CCGCAAGATTCCATACCCA |
