# Supplementary material for: Melatonin Regulates the Neurotransmitter Secretion Disorder Induced by Caffeine Through the Microbiota-Gut-Brain Axis in Zebrafish (Danio rerio)
Source: Front Cell Dev Biol. 2021 May 20;9:678190. doi: 10.3389/fcell.2021.678190 (PMC8172981; doi:10.3389/fcell.2021.678190)
Supplement: Supplementary file 6 [file Table_2.docx]

**Table S2.** Alpha diversity of zebrafish intestinal microbial communities in different treatment groups on day 14.

| **Group** | **Chao1** | **ACE** | **Shannon** | **Simpson** |
| --- | --- | --- | --- | --- |
| Control | 713.01±83.61^#^ | 717.39±91.18^#^ | 4.54±0.16^#^ | 0.84±0.01^#^ |
| Caffeine | 1005.79±159.14^*^ | 1025.06±156.69^*^ | 5.75±0.18^*^ | 0.92±0.01^*^ |
| Melatonin | 983.56±104.76^*^ | 986.74±83.18^*^ | 5.24±0.23^*#^ | 0.87±0.03^#^ |
| Probiotic | 1059.97±71.16^*^ | 1070.67±61.83^*^ | 5.43±0.18^*#^ | 0.90±0.01^*#^ |

**Note:** ^*^, the *p* value between the group and control group was less than 0.05; ^#^, the *p* value between the group and caffeine group was less than 0.05.
