# Supplementary material for: Melatonin Regulates the Neurotransmitter Secretion Disorder Induced by Caffeine Through the Microbiota-Gut-Brain Axis in Zebrafish (Danio rerio)
Source: Front Cell Dev Biol. 2021 May 20;9:678190. doi: 10.3389/fcell.2021.678190 (PMC8172981; doi:10.3389/fcell.2021.678190)
Supplement: Supplementary file 7 [file Table_3.docx]

**Table S3.** Alpha diversity of zebrafish intestinal microbial communities in different treatment groups on day 1.

| **Group** | **Chao1** | **ACE** | **Shannon** | **Simpson** |
| --- | --- | --- | --- | --- |
| Control | 611.71±174.12 | 614.34±181.74 | 5.75±0.71 | 0.91±0.05 |
| Caffeine | 854.15±164.30 | 887.75±193.42 | 4.93±0.88 | 0.86±0.07 |
| Melatonin | 948.00±247.01 | 955.65±245.46 | 5.07±1.11 | 0.85±0.15 |
| Probiotic | 815.59±271.72 | 828.73±277.46 | 4.81±1.05 | 0.84±0.10 |
